# Risk of bias judgements and strength of conclusions in meta-evidence from the Cochrane Colorectal Cancer Group

**DOI:** 10.1186/s13643-019-1001-0

**Published:** 2019-04-08

**Authors:** John Delaney, Rebecca Cui, Alexander Engel

**Affiliations:** 1 Northern Clinical School, Sydney Medical School, University of Sydney, Sydney, Australia; 2 Royal Prince Alfred Hospital, Sydney, Australia; 3 Kolling Institute of Medical Research, Sydney, Australia

**Keywords:** Meta-analysis, Systematic review, Cochrane, Risk of bias, Epidemiologic methods

## Abstract

**Background:**

The Cochrane Collaboration records risk of bias (ROB) judgements on the original studies it analyses. The aim of this review is to perform an audit of all literature produced by the Cochrane Colorectal Cancer Group (CCCG), focusing on whether intervention type has any relationship with ROB and the ability of a review to inform clinical practice.

**Methods:**

The most recent version of every CCCG review from January 2000 to the end of July 2018 was included. Conclusions were categorized as informing clinical practice (I) or not (N). Both I and N categories were divided into firm (F) or tempered (T) based on the definitiveness of their language. ROB judgements were aggregated. Reviews were classed as Medical (M), Surgical (S), Medical & Surgical (MS) or Other (O) based on their intervention, with O reviews then excluded. Data were analyzed in SPSS.

**Results:**

Ninety-five reviews were included, covering 1892 studies. Sixty-two percent (*n* = 59/95) informed clinical practice (I). Thirty-eight percent (*n* = 36/95) did not inform clinical practice (N). Of the N group, 53% (*n* = 19/36) were completely equivocal (firm) while 47% (*n* = 17/36) were moderately so (tempered). In the I group, 46% (*n* = 27/59) gave a conclusion that was firm and 54% (*n* = 32/59) were tempered. Seven thousand five hundred sixty-four cases of bias were assessed. Risk of bias was low in 43%, high in 20% and unclear in 37%. A review that regarded a medical intervention alone was significantly more likely to be comprised of studies with a low risk of bias than a review that included a surgical intervention (*p* < 0.001).

**Conclusion:**

The Cochrane Colorectal Cancer Group finds the risk of bias to be low in less than half of its judgements. A review that included a surgical intervention was less likely to display low risk of bias. Risk of bias was associated with whether a review informed clinical practice, but intervention type was not. Readers of colorectal literature should be cautious when considering original and meta-evidence in this field, particularly where a surgical intervention is assessed.

**Electronic supplementary material:**

The online version of this article (10.1186/s13643-019-1001-0) contains supplementary material, which is available to authorized users.

## Background

The Cochrane Collaboration is an independent [1] global medical research organization [2], well regarded as having a high standard of methodology and rigor [3]. As part of their assessment of original evidence for inclusion in reviews, the Cochrane Collaboration typically assess each study’s risk of bias (ROB), recording a judgement of “low risk”, “high risk” or “unclear risk” across seven standard criteria [4]. The criteria, familiar to many readers, were based on consensus expert review [5] and are intended to focus on the internal validity of studies. Assessment of ROB using the Cochrane tool has been previously noted to have high inter-rater variability [6], and the impact on effect size that a judgement of high or unclear bias in a particular domain has may vary according to the intervention and design of an original study [5]. A judgement of “unclear” may reflect a deficiency in the quality of reporting, rather than poor internal validity of the study. However, Cochrane’s approach is regarded as the gold standard for risk of bias assessment in meta-evidence [7]. Judgements of high *or* unclear risk have been associated with an over-appreciation of effect size [6]. The domains are listed in Table 1. Risk of bias judgements are collated and published in the finished reviews. Cochrane reviews are usually based on randomized control trials and do not consider “real-world data”.

**Table 1:** The Cochrane risk of bias tool

Risk of bias domain	Judgement
Random sequence generation	Selection bias (biased allocation to interventions) due to inadequate generation of a randomized sequence.
Allocation concealment	Selection bias (biased allocation to interventions) due to inadequate concealment of allocations prior to assignment.
Blinding of participants and personnel	Performance bias due to knowledge of the allocated interventions by participants and personnel during the study.
Blinding of outcome assessment	Detection bias due to knowledge of the allocated interventions by outcome assessors.
Incomplete outcome data	Attrition bias due to amount, nature or handling of incomplete outcome data.
Selective reporting	Reporting bias due to selective outcome reporting.
Other bias	Bias due to problems not covered elsewhere in the table.

Cochrane’s colorectal cancer group is the CCCG, the Cochrane Colorectal Cancer Group [8]. Each review published by the CCCG typically includes a recording of the ROB judgements of the relevant group of original studies. The overall picture of bias within the literature assessed by the CCCG has yet to be aggregated. This paper will collect and analyze all ROB judgements published by the CCCG, giving an overview of the risk of bias found in colorectal original research. The combined data will provide a view of the quality of a sample of colorectal literature over time, and a snapshot of its current status. It will also offer insight into the level of clinical utility of scientific publications within the colorectal domain; that is, when Cochrane reviews the colorectal literature, how often is it able to inform clinical practice?

As a subset of surgical intervention, laparoscopic surgery presents an example of the surgical research dilemma; technology precedes evidence, and the barriers to surgeons using new devices are low [9]. The learning curve of a new surgical implement and the pre-existing challenges of surgical research make robust conclusions about best practice difficult, particularly in the early phase of implementation [10]. This is in a commercial setting of high public demand and great marketing potential for new surgical technologies, as may also be seen currently with the popularity of robotic surgery [11, 12]. Laparoscopic surgery (“key-hole” surgery) became widespread *prior* to significant evidence demonstrating its superiority over open approaches [13] and even now there remain some areas of controversy [14]. Subgroup analysis on laparoscopic papers from within the CCCG output was planned for this review as a means of assessing the meta-evidence support for this surgical technique.

## Methods

### Literature search

In collaboration with the CCCG, a list of all of that group’s reviews from January 2000 to the end of July 2018 was acquired. The recorded ROB judgements for each study were also provided. The provided database was compared by two independent reviewers (JD and RC) with reviews retrieved from the Cochrane Library to check accuracy.

### Definitions

Risk of bias judgements are defined in the Cochrane Collaboration handbook [4]. Conclusion type was classified as “informs clinical practice—firm” (I-F), “informs clinical practice—tempered” (I-T), “does not inform clinical practice—tempered” (N-T) and “does not inform clinical practice—firm” (N-F). The definitions for these categories are outlined in Table 2 and have been described and used previously [15].

**Table 2:** Categories of conclusion type

Conclusion type	Criteria
Informs clinical practice*—*firm	A conclusion that makes a recommendation for practice (positive or negative), with minimal or no caveats.
Informs clinical practice*—*tempered	A conclusion that makes a recommendation for practice, but places significant caveats on that recommendation.
Does not inform clinical practice*—*tempered	A conclusion that is unable to make a recommendation but suggests that a recommendation might be possible soon based on an emerging trend or underlying theory.
Does not inform clinical practice*—*firm	A conclusion that is unable to make a recommendation and is completely uncertain. There may be no evidence at all, or of too poor a quality, or the evidence may be contradictory.

Reviews were classed as Medical (M), Surgical (S), Medical & Surgical (MS) or Other (O) based on the intervention. An M paper considered an intervention that was exclusively medical, whereas an S paper examined an intervention that was exclusively surgical in nature. An MS paper was one where a surgical intervention was assessed in the setting of medical intervention or vice versa, for example, *Epidural local anesthetics* versus *opioid-based analgesic regimens for postoperative gastrointestinal paralysis, vomiting and pain after abdominal surgery* [16]*.* A review that did not assess an intervention or incorporated a therapy that was neither surgical nor medical (for instance, radiotherapy) was classified as “Other” (O).

### Inclusion and exclusion criteria

Included papers were systematic reviews and meta-analyses produced by the Cochrane Colorectal Cancer Group from January 2000 to the end of July 2018 that were classified as M, MS or S. Where more than one version of a review had been produced, the most up-to-date version was preferred. Reviews that were classified as O (that is, reviews that considered no intervention, or an intervention that was neither surgical nor medical) were a priori excluded from this analysis to facilitate comparison of surgical and medical interventions.

### Data extraction

ROB judgements were extracted from each included Cochrane review. Conclusions and intervention type (M, S, MS or O) were scored independently by JD and RC. Each CCCG review was categorized based on its ability to inform clinical practice, using a standardized matrix (Table 2). Any disagreements were resolved by discussion to arrive at a consensus. Collation of data was performed in Microsoft Excel [17].

A subgroup of reviews concerning laparoscopic interventions was isolated and assessed (17 reviews). For visual comparison, a graphical representation of the commentary made on evidence within the conclusions of those reviews (a “word cloud”) was generated using Microsoft Word.

### Statistical analysis

Analysis of data was performed using SPSS v24 [18]. Inter-observer agreement was assessed using weighted kappa (*κ*) [19]. Data that were categorical were analyzed was via cross tabulation with chi-square. A two-tailed distribution with an alpha level of 0.05 was used, with a Bonferroni correction where required. The means of continuous data were compared using one-way ANOVA with planned contrasts. An alpha level of 0.05 was used, again with a Bonferroni correction where required.

## Results

**Fig. 1:**
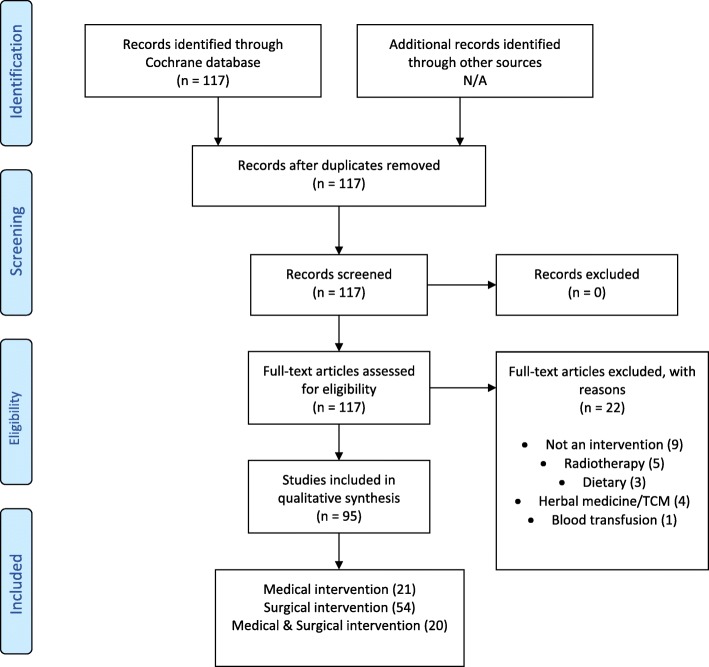
PRISMA flow diagram

The data provided by the CCCG included 117 unique Cochrane reviews. Twenty-two reviews were excluded from our audit; 9 regarded diagnostics rather than intervention, 5 reviews concerned radiotherapy, 4 examined herbal therapy, 3 regarded dietary modifications and 1 considered the impact of a perioperative blood transfusion (see Appendix 1 and 2). A PRISMA flow diagram may be found in Fig. 1. The PRISMA checklist is available as an Additional file 1. The 95 included reviews covered 1892 original studies. This created 13,244 possible cases of bias to be judged (7 bias categories across 1892 studies).

**Fig. 2:**
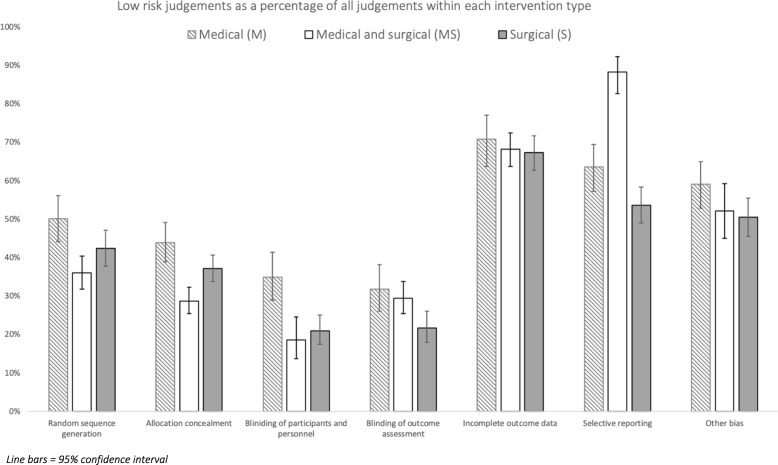
Low-risk judgements as a percentage of all judgements within each intervention type

The CCCG made a combined total of 7564 judgements across the seven ROB categories. In 5680 instances, a ROB judgement was not recorded by the Cochrane Group (for example, when a review provided a judgement for some but not all of the ROB criteria). Overall, bias was judged as low 43% of the time (*n* = 3291), high 20% of the time (*n* = 1490) and unclear 37% of the time (*n* = 2783). Bias judgements for each intervention type may be found in Table 3. A chi-square test was performed, and a significant relationship found between the intervention type and the likelihood of a study to show differing bias (chi-square, *df* 4, 41.696, *p* < 0.001). Comparison of individual groups using chi-square with a Bonferroni correction (*α* = 0.0024) revealed M to be more likely than S and MS to have studies with a low risk of bias (M = 50% > S = 42%, *p* < 0.001), (M = 50% > MS = 42%, *p* = 0.002). No difference was found in the likelihood of a low risk of bias when comparing S with MS (*p* = 0.831). Figure 2 displays low-risk judgements by intervention as a percentage of judgements made across Cochrane’s seven categories. Assessment of the likelihood of high-risk judgements showed S reviews to be more likely than M reviews to show a high risk of bias (S = 21% v M = 16%, *p* < 0.001). MS reviews were also more likely than M to display high risk (MS = 20% > M = 16%, *p* < 0.001). There was no difference in the likelihood of a high risk of bias between S and MS reviews (*p* = 0.2294).

**Table 3:** Bias judgements by intervention type

	Reviews	Studies	Patients	Total bias judgements made	Low risk of bias (% of judgements made)	High risk of bias (% of judgements made)	Unclear risk of bias (% of judgements made)	Informs clinical practice*—*firm (% of reviews)	Informs clinical practice*—*tempered (% of reviews)	Does not inform clinical practice*—*tempered (% of reviews)	Does not inform clinical practice*—*firm (% of reviews)
All	95	1892	525,927	7564	3291 (43)	1490 (20)	2783 (37)	32 (34)	27 (28)	17 (18)	19 (20)
M	21	368	105,200	1732	863 (50)	278 (16)	591 (34)	15 (71)	2 (10)	1 (5)	3 (14)
S	54	834	198,514	3248	1348 (42)	694 (21)	1206 (37)	11 (20)	20 (37)	10 (19)	13 (24)
MS	20	690	221,190	2584	1080 (42)	518 (20)	986 (38)	6 (30)	5 (25)	6 (30)	3 (15)

*M* medical, *S* surgical, *MS* medical and surgical

M had the greatest percentage of low-risk judgements across all risk-of-bias categories with the exception of selective reporting, where MS had the greatest percentage. When comparing M with S using chi-square with a Bonferroni correction (*α* = 0.0024), M group reviews were found to be significantly more likely to be comprised of studies with a low risk of bias than S reviews in the category of blinding of participants and personnel (M = 35% > S = 21%, *p* < 0.001). No difference was found in the likelihood to display low risk in random sequence generation (M = 50%, S = 42%, *p* = 0.049), allocation concealment (M = 44%, S = 37%, *p* = 0.035), blinding of outcome assessment (M = 32%, S = 22% *p* = 0.007), incomplete outcome data (M = 71%, S = 67%, *p* = 0.439), selective reporting (M = 64%, S = 54%, *p* = 0.0145) and other bias (M = 59%, M = 51%, *p* = 0.035). S reviews were more likely to display a high risk of bias than M reviews in the domains of random sequence generation (S = 12% > M = 2%, *p* < 0.001) and AC (S = 11% > M = 2%, *p* < 0.001). No difference was found in the chance of a high-risk judgement in the areas of blinding of participants and personnel (S = 41%, M = 44%, *p* = 0.559), blinding of outcome assessment (S = 48%, M = 45%, *p* = 0.616), incomplete outcome data (S = 17%, M = 8%, *p* = 0.005), selective reporting (S = 13%, M = 7%, *p* = 0.013) and other bias (S = 16%, M = 15%, *p* = 0.659).

Comparing M with MS, M reviews were significantly more likely to be comprised of studies displaying low bias in random sequence generation (M = 50% > MS = 36%, *p* < 0.001), allocation concealment (M = 44% > MS = 29%, *p* < 0.001) and blinding of participants and personnel (M = 32% > MS = 19%, *p* < 0.001). MS was more likely than M to display low bias in the domain of selective reporting (MS = 88% > M = 64%, *p* < 0.001). Differences were not found between MS and M groups in the chance of a low-risk judgement in blinding of outcome assessment (M = 32%, MS = 29%, *p* = 0.534), incomplete outcome data (M = 71%, MS = 68%, *p* = 0.562) and other (M = 59%, MS = 52%, *p* = 0.173). MS reviews were more likely to have a judgement of high risk in the areas of allocation concealment (MS = 20% > M = 2%, *p* < 0.001) and incomplete outcome data (MS = 27% > M = 8%, *p* < 0.001). M was more likely to show high risk in blinding of participants and personnel (M = 44% > MS = 24%, *p* < 0.001). No difference in high-risk judgements were found in the areas of random sequence generation (M = 2%, MS = 1%, *p* = 0.764), blinding of outcome assessment (M = 45%, MS = 45%, *p* = 0.87), selective reporting (M = 7%, MS = 1%, *p* = 0.006) or other bias (M = 15%, MS = 5%, *p* = 0.0011).

Contrasting MS with S, MS reviews were significantly more likely to come from original studies with low risk of bias judgements in the categories of selective reporting (MS = 88% > S = 54%, *p* < 0.001). S reviews were more likely to have a greater proportion of low-risk judgements in the domain of allocation concealment (S = 37% > MS = 29%, *p* < 0.001). Differences between the two interventions were non-significant in random sequence generation (MS = 36%, S = 42%, *p* = 0.055), blinding of participants and personnel (MS = 19%, S = 21%, *p* = 0.038), blinding of outcome assessment (MS = 29%, S = 22%, *p* = 0.524), incomplete outcome data (MS = 68%, S = 67%, *p* = 0.826) and other bias (MS = 52%, S = 51%, *p* = 0.721). S reviews were more likely to have studies with a high risk of bias in the areas of random sequence generation (S = 12% > S = 1%, *p* < 0.001), selective reporting (S = 13% > MS = 1%, *p* = 0.001), and other bias (S = 16% > MS = 5%, *p* < 0.001). MS was more likely to have a high risk of bias judgement in allocation concealment (MS = 20% > S = 11%, *p* < 0.001) and incomplete outcome data (MS = 27% > S = 17%, *p* < 0.001). No difference was found in blinding of outcome assessment (MS = 45%, S = 48%, *p* = 0.411).

Overall, the reviews firmly informed clinical practice (I-F) 34% of the time (*n* = 32/95) and informed practice in a tempered fashion (I-T) 28% of the time (*n* = 27/95). A conclusion that did not inform clinical practice but was tempered (N-T) was made 18% of the time (*n* = 17/95) and a conclusion that did not inform clinical fashion and was firm (N-F) was made 20% of the time (*n* = 19/95). There was substantial [20] inter-observer agreement on the conclusiveness of the reviews (weighted kappa = 0.622). Initial disagreements were primarily found in the differentiation between firm and tempered conclusions; comparison of “informs clinical practice” vs “does not inform clinical practice” decisions revealed a kappa of 0.753. A consensus was achieved for each review.

**Fig. 3:**
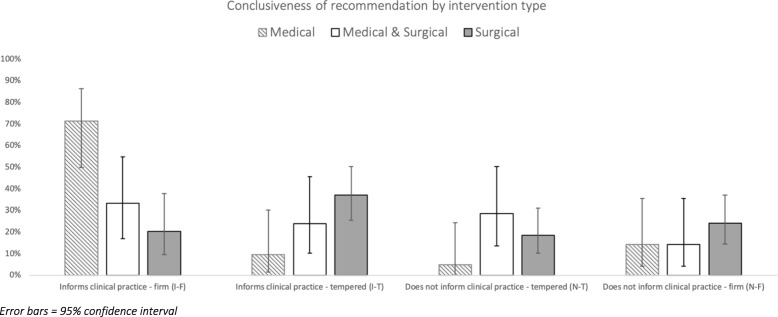
Conclusiveness of recommendation by intervention type

A chart of the conclusiveness of reviews by intervention is shown in Fig. 3. There was a significant difference between the groups when assessed using chi-square (*df* 6, 20.274, *p* = 0.002). Comparison of individual groups using chi-square with a Bonferroni correction (*α* = 0.004) was performed. M reviews were significantly more likely to provide conclusion that firmly informed clinical practice than S (M = 71% > S = 20%, *p* < 0.001) but not MS (M = 71%, MS = 30%, *p* = 0.0294). Reviews informed clinical practice (regardless of whether firm or tempered) in 81% of M reviews, 55% of MS reviews and 57% of S reviews, but there was no significant difference between these groups (M v S *p* = 0.066, M v MS *p* = 0.1, S v MS *p* = 1).

The risk of bias groups and their relationship to conclusion type were examined via chi-square, with a significant difference found between the groups (chi-square, *df* 6, 311.465, *p* < 0.001). Comparison of I and N groups using chi-square with a Bonferroni correction (*α* = 0.0018) revealed a significant difference in the likelihood of input studies being judged as having a low risk of bias between them (I = 46% > N = 35%, *p* < 0.001).

**Fig. 4:**
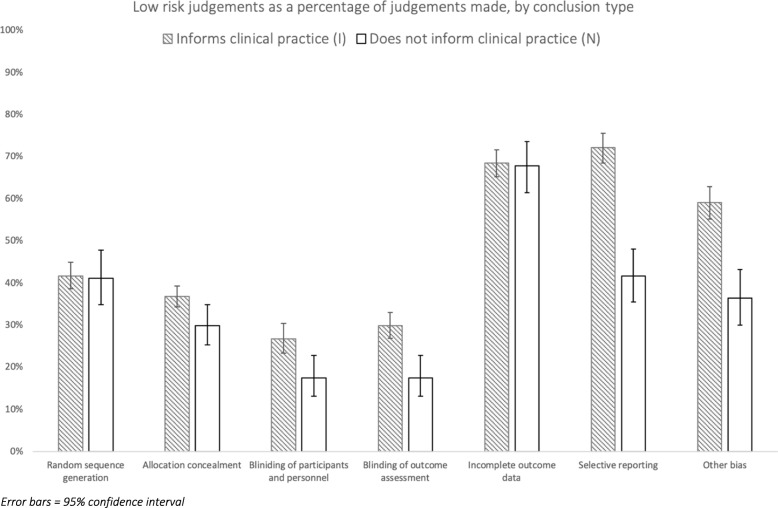
Low-risk judgements as a percentage of judgements made, by conclusion type

Chi-square with a Bonferroni correction (*α* = 0.0018) was used to examine whether there was any association between the seven risk of bias categories and a review’s likelihood to inform clinical practice. Risk of bias domains that had a significant association between low-risk judgements and conclusion type were blinding of outcome assessment (I = 42% low risk > N = 17% low risk, *p* < 0.001), selective reporting (I = 72% > N = 42%, *p* < 0.001) and other bias (I = 59% > N = 36%, *p* > 0.001). There was not a significant association found between random sequence generation (I = 42%, N = 41%, *p* = 0.939), allocation concealment (I = 37%, N = 30%, *p* = 0.017), blinding of participants and personnel (I = 27%, N = 17%, *p* = 0.004) and incomplete outcome data (I = 68%, N = 68%, *p* = 0.871). A chart of risk of bias by ability to inform clinical practice is shown in Fig. 4.

**Fig. 5:**
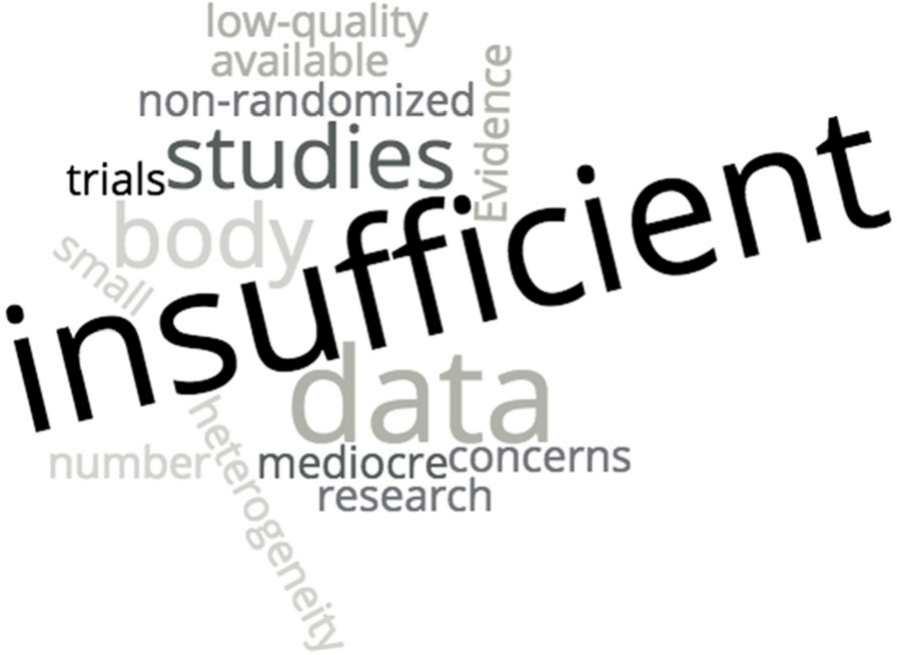
Word cloud of commentary on evidence within laparoscopic review conclusions

For interest, the conclusions of a subgroup of reviews that considered laparoscopic interventions were assessed, shown in Table 4. Seventeen reviews were found. The modal recommendation was I-T in 8 (47%), followed by N-F in 5 (29%), N-T in 2 (12%) and I-F in 2 (12%). A “word cloud” made using descriptions of evidence quality from the conclusions of each of these reviews may be seen in Fig. 5.

**Table 4:** Reviews assessing laparoscopic interventions

Review title	Studies	Patients	Rec.	RSG (% low risk)	AC (% low risk)	BPP (% low risk)	BOA (% low risk)	IOD (% low risk)	SR (% low risk)	OB (% low risk)	Evidence commentary
Closure methods for laparotomy incisions for preventing incisional hernias and other wound complications	55	19,174	I-T	25	27	22	N/A	69	93	84	Based on this moderate-quality body of evidence…
Closure methods of the appendix stump for complications during laparoscopic appendectomy	8	850	N-F	50	25	0	13	63	0	75	Evidence is insufficient at present…
Energy source instruments for laparoscopic colectomy	6	515	N-F	50	83	N/A	N/A	100	100	67	With the current evidence it is not possible…
Gases for establishing pneumoperitoneum during laparoscopic abdominal surgery	7	340	N-T	29	29	57	71	57	71	57	No comment
Hand assisted laparoscopic surgery versus conventional laparoscopy for colorectal surgery	3	189	I-T	N/A	67	0	0	67	67	N/A	No comment
Heated insufflation with or without humidification for laparoscopic abdominal surgery	22	1428	I-F	55	59	74	74	82	86	86	No comment
Laparoscopic techniques versus open techniques for inguinal hernia repair	40	6205	I-T	N/A	48	N/A	N/A	N/A	N/A	N/A	No comment
Laparoscopic versus open resection for sigmoid diverticulitis	3	392	N-F	67	67	0	0	33	67	N/A	evidence to support or refute … is insufficient.
Laparoscopic versus Open surgery for small bowel Crohn’s disease	4	249	I-T	0	50	0	0	N/A	100	0	No comment
Laparoscopic versus open surgery for suspected appendicitis	66	7148	I-T	37	63	13	13	49	15	57	In spite of the mediocre quality of the available research data, we would generally recommend…
Laparoscopic versus open surgery in small bowel obstruction	0	0	N-F	N/A	N/A	N/A	N/A	N/A	N/A	N/A	..data from retrospective clinical controlled trials..
Laparoscopic versus open surgical techniques for ventral or incisional hernia repair	10	880	N-T	60	70	0	0	50	20	30	No comment
Laparoscopic versus open total mesorectal excision for rectal cancer	48	4067	I-T	N/A	0	N/A	N/A	N/A	N/A	N/A	Based on evidence mainly from non-randomized studies…
Long-term results of laparoscopic colorectal cancer resection	12	3346	I-T	N/A	58	N/A	N/A	N/A	N/A	N/A	No comment
Open versus laparoscopic (assisted) ileo pouch anal anastomosis for ulcerative colitis and familial adenomatous polyposis	11	673	I-T	N/A	9	N/A	N/A	N/A	N/A	N/A	No comment
Short term benefits for laparoscopic colorectal resection	24	3474	I-F	N/A	28	N/A	N/A	N/A	N/A	N/A	No comment
Transabdominal pre-peritoneal (TAPP) vs totally extraperitoneal (TEP) laparoscopic techniques for inguinal hernia repair	10	19,738	N-F	N/A	N/A	N/A	N/A	N/A	N/A	N/A	There is insufficient data.

*I-F* informs clinical practice*—*firm, *I-T* informs clinical practice*—*tempered, *N-T* does not inform clinical practice*—*tempered, *N-F* does not inform clinical practice*—*firm, *RSG* random sequence generation, *AC* allocation concealment, *BPP* blinding of participants and personnel, *BOA* blinding of outcome assessors, *IOD* incomplete outcome data, *SR* selective reporting, *OB* other bias, *N/A* no judgements made

## Discussion

This review has gathered previously made judgements on the risk of bias from a large sample of original research within the colorectal field and combined them, forming a portrait of the risk of bias within the discipline generally. We have then added to this data new judgements regarding the type of intervention studied and the clinical relevance of the meta-evidence produced. Using this approach, a view of the quality of data input *and* data output within colorectal science may be formed. The importance of these results relates to the defining characteristic of intervention-based medical research: the drive to improve patient outcomes. Studies that exhibit a high risk of bias lead to meta-evidence that is less able to guide clinical outcomes, together forming clinical “noise”, from which clinicians are tasked with separating the “signal”. The results of this review illustrate the benchmarks within colorectal science of “signal” and “noise”, and whether there are any associated factors that will help guide the production of useful original and combined evidence.

When the Cochrane Colorectal Cancer Group examines the risk of bias in original studies within colorectal science, it found the risk of bias to be low in the minority of cases. Notably, many of the studies assessed by the CCCG do not specifically address questions surrounding colorectal cancer per se (for instance, “*Cisapride for Intestinal Constipation*” [21], or “*Antibiotics for uncomplicated diverticulitis*” [22]), but rather provide an overview of colorectal science in general, with an inclination towards cancer research. The opinion of the Cochrane group was that the risk of bias within this sample was high or unclear in greater than half of cases. If we consider risk of bias judgements that could possibly have been made but were not to be “unclear” the proportion of low-risk judgements to high or unclear drops further. This review suggests that the minority rate of low-risk judgements across all of the CCCG should be noted by researchers and readers in the colorectal discipline. Efforts to minimize the risk of bias within this field should be considered, led by feedback from meta-evidence.

There was an association found between the intervention type and the level of low risk of bias judgements made. Studies concerning a medical intervention (M) were significantly more likely to display low risk of bias than studies concerning a surgical intervention only, or studies with a surgical and medical intervention. Studies that assessed only a surgical intervention were judged to have a low risk of bias at a rate that was not significantly different to studies that assessed a medical *and* surgical intervention. S reviews and MS reviews were significantly more likely than M reviews to have high-risk judgements. This may suggest that the addition of a surgical intervention may decrease the amount of bias protection inherent in the assessment of a medical intervention.

Across all interventions, the rate of low-risk judgements was 50% or less in random sequence allocation, allocation concealment and blinding of outcome assessment and participants and personnel. That the randomization, concealment and blinding for all studies within the CCCG reviews were judged as having high or unclear risk of bias in more than half of cases is cause for reflection. Previous research has demonstrated that high-risk judgements from the Cochrane risk of bias tool are associated with increased effect sizes [6, 23]. It should be noted that within this sample over 5000 risk of bias judgements that could have been made were not, which potentially dilutes the generalizability and impact of this finding.

Despite a significant difference in low risk of bias findings between M and S overall, analysis of each domain did not reveal a deeper pattern, though a significant difference was found in the blinding of participants and personnel, and a near-significant difference seen in the blinding of outcome assessors. Perhaps surprisingly, the rate of high-risk judgements made regarding blinding was not significantly different between M and S reviews. Both M and S reviews were judged to be high risk for binding in nearly half of all cases. Difficulty in adequately blinding is the nature of the surgical intervention and remains a systemic challenge to rigorous science in surgery. However, the result for the M group suggests that where practical, medical colorectal studies may benefit from paying particular attention to the risk of bias due to inadequate blinding.

M and MS groups were significantly different across a range of bias categories, with M being more likely than MS to have a low risk of bias in random sequence allocation, allocation concealment and blinding of participants and personnel, and MS being more likely to display low risk in selective reporting. As discussed, the inclusion of a surgical intervention in a study makes participant and personnel blinding difficult, which may explain why MS reviews were less likely than M to have a low risk of bias in this domain. Errors in reporting may explain the difference in random sequence generation, as MS was no more likely to be judged as high risk in this area, suggesting the discrepancy to be related to a large proportion of “unclear” judgements. Within allocation concealment, however, MS reviews were more likely to have less low-risk judgements *and* they were significantly more likely to have a high-risk judgement. The increased exposure to bias from poor allocation concealment within MS reviews is also present when contrasting MS with S reviews. This result is attributable to the impact of a single MS review, “*Antimicrobial prophylaxis for colorectal surgery*” [24], which had a high-risk judgement rate for allocation concealment of 45% and provided 115 of the 133 allocation concealment judgements made.

Intervention type was not associated with whether or not a review would be able to inform clinical practice. The likelihood that a review will make a conclusion that informs clinical practice is similar between surgical, medical and combined medical and surgical meta-evidence, despite the fact that studies that incorporate a surgical intervention are more likely to display high or unclear bias. In contrast, there was an association between whether a review informed clinical practice and the risk of bias. M reviews were better protected against bias than S and MS reviews but were no less likely to inform clinical practice. Where a conclusion did inform practice, the likelihood that it would be firm, rather than tempered, was significantly different M and S reviews, but not M and MS. Reviews that examined a surgical intervention exclusively were less confident about their conclusions but were willing to inform practice. It is speculative, but this may suggest that the threshold for a clinical recommendation within surgical evidence is lower than that in medical evidence. Readers of these reviews should be conscious of the GRADE quality assessments that accompany modern Cochrane reviews [25] and consider all low-quality clinical recommendations cautiously. It may be possible that a new weighting metric for meta-evidence, which combines a weighted risk of bias quality score with the cohort size of each study, may deliver meta-analysis that better accounts for varying input quality.

The subgroup analysis of laparoscopic surgery, including the “word cloud”, shows a negative view of the quality of the input studies and a lack of confidence regarding the evidence. In spite of this, a conclusion that informs clinical practice is made in nearly half of all cases, albeit tempered. The following quoted conclusion, regarding the use of laparoscopic or open techniques in the management of suspected appendicitis, one of surgery’s most common ailments, illustrates the challenge surgeons face; “In spite of the mediocre quality of the available research data, we would generally recommend to use laparoscopy…in patients with suspected appendicitis unless laparoscopy itself is contraindicated or not feasible” [26].

These findings suggest that the original input studies informing meta-evidence within surgery may be inherently biased in a way that makes evidence synthesis in this field less applicable. In the face of this, evidence-based surgery remains elusive. Novel ways of thinking about surgical research may need to be employed. This is not a new revelation [27], but perhaps there are technological advances now that afford surgical research an opportunity not present before. In particular, cloud-based “big data” collection and machine learning analysis may be useful; an approach where high-fidelity patient and practitioner data are automatically recorded for analysis across multiple centres may provide a better avenue to approximate the real-world impact of surgical interventions.

### Strengths and limitations

The strengths of this “review of reviews” are the large number of Cochrane reviews included in our assessment and the dual independent extraction of data. Cochrane is viewed as the gold standard of systematic reviews. Assessment of risk of bias is standardized across Cochrane trials, which enables comparison. Our methods and definitions have been clearly outlined.

The limitations of this study include the restriction of data to Cochrane reviews only, introducing the possibility of selection bias. The CCCG did not record a judgement in over 5000 of the instances where it could have, creating potential bias. The risk of bias judgements recorded by Cochrane are subjective and open to bias. Likewise, the judgements made by the authors of this review regarding the conclusiveness of each of the Cochrane reviews are subjective.

## Conclusion

The findings of this study highlight a need for more detailed reporting and a greater degree of methodological rigor within original colorectal research. Although the type of intervention was associated with a higher risk of bias, it was not associated with the likelihood of a review to inform clinical practice. Surgical studies, in particular, are prone to a higher degree of bias risk and must be interpreted with caution; a review that included a surgical intervention was likely to have a higher risk of bias but was just as likely to inform clinical practice. This may be reflective of the systemic challenges of surgical research.

### Additional file

Additional file 1: Prisma 2009 checklist. (DOC 64 kb)

## Appendix

**Table 5:** Excluded reviews

Review	Year	Reason for exclusion
Blood CEA levels for detecting recurrent colorectal cancer	2015	Non-interventional
Chinese medical herbs for chemotherapy side effects in colorectal cancer patients	2005	Herbal
Chromoscopy versus conventional endoscopy for the detection of polyps in the colon and rectum	2016	Non-interventional
Concomitant hyperthermia and radiation therapy for treating locally advanced rectal cancer	2009	Radiotherapy
Dietary calcium supplementation for preventing colorectal cancer and adenomatous polyps	2008	Dietary
Dietary fiber for the prevention of recurrent colorectal adenomas and carcinomas	2017	Dietary
Dietary flavonoid for preventing colorectal neoplasms	2012	Dietary
Flexible sigmoidoscopy versus fecal occult blood testing for colorectal cancer screening in asymptomatic individuals	2013	Non-interventional
Follow-up strategies for patients treated for non-metastatic colorectal cancer	2007	Non-interventional
Herbal medicines for advanced colorectal cancer	2012	Herbal
Narrow band imaging versus conventional white light colonoscopy for the detection of colorectal polyps	2012	Non-interventional
Oral traditional Chinese medication for adhesive small bowel obstruction	2012	Herbal
Perioperative blood transfusions and recurrence of colorectal cancer	2012	Blood transfusion
Pre-operative radiotherapy and curative surgery for the management of localized rectal carcinoma	2007	Radiotherapy
Preoperative chemoradiation versus radiation alone for stage II and III resectable rectal cancer	2013	Radiotherapy
Radiofrequency ablation in the treatment of liver metastases from colorectal cancer	2012	Radiotherapy
Screening for colorectal cancer using the fecal occult blood test, Hemoccult	2007	Non-interventional
Selective internal radiation therapy for liver metastases from colorectal cancer	2009	Radiotherapy
Virtual reality simulation training for health professions trainees in gastrointestinal endoscopy	2012	Non-interventional
Workload and surgeon’s specialty for outcome after colorectal cancer surgery	2012	Non-interventional
Traditional Chinese Medicine herbs for stopping bleeding from hemorrhoids	2010	Herbal
Transparent cap colonoscopy versus standard colonoscopy to improve caecal intubation	2012	Non-interventional

**Table 6:** Included reviews

Review	Year	Author	Intervention	Conclusion
Abdominal drainage to prevent intra-peritoneal abscess after open appendectomy for complicated appendicitis	2015	Nelson RL, et al.	Surgical	N-F
Adjuvant chemotherapy for small intestine adenocarcinoma	2007	Singhal N, Singhal D	Medical	N-F
Adjuvant Therapy for completely resected Stage II Colon Cancer	2008	Figueredo A, et al.	Medical & Surgical	I-T
Analgesia in patients with acute abdominal pain	2011	Manterola C, et al.	Medical	I-F
Anti-angiogenic therapies for metastatic colorectal cancer	2009	Wagner ADADW, et al.	Medical	I-F
Antibiotic prophylaxis for hernia repair.	2012	Sanchez-Manuel FJ, et al.	Medical & Surgical	N-T
Antibiotic regimens for secondary peritonitis of gastrointestinal origin in adults	2005	Wong PF, et al.	Medical	I-F
Antibiotics for uncomplicated diverticulitis	2012	Shabanzadeh DM, Wille-Jørgensen P	Medical	N-T
Antibiotics versus placebo for prevention of postoperative infection after appendicectomy.	2005	BR Andersen, et al.	Medical & Surgical	I-F
Antimicrobial prophylaxis for colorectal surgery	2014	Nelson RL, et al.	Medical & Surgical	I-F
Appendectomy versus antibiotic treatment for acute appendicitis	2011	Wilms IMHA, et al.	Medical & Surgical	I-T
Cesarean delivery for the prevention of anal incontinence	2010	Nelson RL, et al.	Surgical	I-F
Chewing gum for postoperative recovery of gastrointestinal function	2015	Short V, et al.	Surgical	N-T
Cisapride for Intestinal Constipation	2009	Aboumarzouk OM, et al.	Medical	I-F
Closure methods for laparotomy incisions for preventing incisional hernias and other wound complications	2017	Patel SV, et al.	Surgical	I-T
Closure methods of the appendix stump for complications during laparoscopic appendectomy	2017	Mannu GS, et al.	Surgical	N-F
Colorectal stents for the management of malignant colonic obstructions	2011	Sagar J	Surgical	I-T
Combination chemotherapy versus single-agent chemotherapy during preoperative chemoradiation for resectable rectal cancer	2015	Resende HM, et al.	Medical & Surgical	N-F
Conventional versus LigaSure hemorrhoidectomy for patients with symptomatic Hemorrhoids	2009	Nienhuijs SW, et al.	Surgical	I-T
Covering ileo- or colostomy in anterior resection for rectal carcinoma	2010	Montedori A, et al.	Surgical	I-T
Curative surgery for obstruction from primary left colorectal carcinoma- Primary or staged resection?	2004	De Salvo GL, et al.	Surgical	N-F
Daikenchuto for reducing postoperative ileus in patients undergoing elective abdominal surgery	2018	Hoshino N, et al.	Medical & Surgical	N-F
Duration of adjuvant chemotherapy for patients with non-metastatic colorectal cancer	2010	Des Guetz G, et al.	Medical	I-F
Early enteral nutrition within 24 h of colorectal surgery versus later commencement of feeding for postoperative complications	2006	Andersen HK, et al.	Surgical	N-T
Early versus delayed appendicectomy for appendiceal phlegmon or abscess	2017	Cheng Y, et al.	Surgical	N-F
Energy source instruments for laparoscopic colectomy	2011	Tou S, et al.	Surgical	N-F
Epidermal growth factor receptor (EGFR) inhibitors for metastatic colorectal cancer	2017	Chan DLH, et al.	Medical	I-F
Epidural local anesthetics versus opioid**-**based analgesic regimens for postoperative gastrointestinal paralysis, vomiting and pain after abdominal surgery	2016	Guay J, et al.	Medical & Surgical	I-F
Fast track surgery versus conventional recovery strategies for colorectal surgery	2011	Spanjersberg WR, et al.	Surgical	N-T
Fluoropyrimidine-HAI (hepatic arterial infusion) versus systemic chemotherapy (SCT) for unresectable liver metastases from colorectal cancer	2009	Mocellin S, et al.	Medical	I-F
Gases for establishing pneumoperitoneum during laparoscopic abdominal surgery	2013	Cheng Y, et al.	Surgical	N-T
Hand assisted laparoscopic surgery versus conventional laparoscopy for colorectal surgery	2010	Spanjersberg WR, et al.	Surgical	I-T
Heated insufflation with or without humidification for laparoscopic abdominal surgery	2016	Birch DW, et al.	Surgical	I-F
Heparins and mechanical methods for thromboprophylaxis in colorectal surgery	2004	Wille-Jørgensen P, et al.	Medical & Surgical	I-F
Hepatic artery adjuvant chemotherapy for patients having resection or ablation of colorectal cancer metastatic to the liver	2006	Nelson RL, Freels S	Medical & Surgical	N-T
Histamine type 2 receptor antagonists as adjuvant treatment for resected colorectal cancer	2012	Deva S, Jameson M	Medical	I-T
Ileostomy or colostomy for temporary decompression of colorectal anastomosis	2007	Güenaga KF, et al.	Surgical	N-T
Incision and drainage of perianal abscess with or without treatment of anal fistula	2010	Malik AI, et al.	Surgical	I-F
Interventions for anal canal intraepithelial neoplasia	2012	Macaya A, et al.	Surgical	N-F
Intra-abdominal drains for the prophylaxis of anastomotic leak in elective colorectal surgery	2004	Rolph R, et al.	Surgical	N-F
Intra-peritoneal prophylactic agents for preventing adhesions and adhesive intestinal obstruction after non-gynecological abdominal surgery	2009	Kumar S, et al.	Medical & Surgical	N-T
Irinotecan chemotherapy combined with fluoropyrimidines versus irinotecan alone for overall survival and progression-free survival in patients with advanced and/or metastatic colorectal cancer	2016	Wulaningsih W, et al.	Medical	N-F
Lactulose versus Polyethylene Glycol for Chronic Constipation	2010	Lee-Robichaud H, et al.	Medical	I-F
Laparoscopic techniques versus open techniques for inguinal hernia repair	2003	Willaert W, et al.	Surgical	I-T
Laparoscopic versus open resection for sigmoid diverticulitis	2017	Abraha I, et al.	Surgical	N-F
Laparoscopic versus open surgery for small bowel Crohn’s disease	2011	Dasari BVM, et al.	Surgical	I-T
Laparoscopic versus open surgery in small bowel obstruction	2010	Cirocchi R, et al.	Surgical	N-F
Laparoscopic versus open surgery for suspected appendicitis	2010	Sauerland S, et al.	Surgical	I-T
Laparoscopic versus open surgical techniques for ventral or incisional hernia repair	2011	Sauerland S, et al.	Surgical	N-T
Laparoscopic versus open total mesorectal excision for rectal cancer	2014	S Vennix, et al.	Surgical	I-T
Lateral pararectal versus transrectal stoma placement for prevention of parastomal herniation	2013	Hardt J, et al.	Surgical	N-F
Laxatives for the treatment of hemorrhoids.	2005	Alonso-Coello P, et al.	Medical	I-F
Long-term results of laparoscopic colorectal cancer resection	2008	E Kuhry, et al.	Surgical	I-T
Management for intussusception in children	2017	Gluckman S, et al.	Medical & Surgical	N-T
Mechanical bowel preparation for elective colorectal surgery	2011	Güenaga KF, et al.	Surgical	N-T
Mesalamine (5**-**ASA) for the prevention of recurrent diverticulitis	2017	Carter F, et al.	Medical	N-F
Mesh fixation with glue versus suture for chronic pain and recurrence in Lichtenstein inguinal hernioplasty	2017	Sun P, et al.	Surgical	I-T
Nitrous Oxide for Colonoscopy	2011	Aboumarzouk OM, et al.	Surgical	I-T
Nonsteroidal anti-inflammatory drugs (NSAID) and aspirin for preventing colorectal adenomas and carcinomas	2004	Asano TK, McLeod RS	Medical	I-F
Nonsurgical therapy for anal fissure	2012	Nelson RL, et al.	Medical	I-F
Non-resection versus resection for an asymptomatic primary tumor in patients with unresectable Stage IV colorectal cancer	2012	Cirocchi R, et al.	Medical & Surgical	I-T
Open Mesh versus non-Mesh for groin hernia repair	2001	Scott N, et al.	Surgical	I-T
Open Preperitoneal Techniques versus Lichtenstein Repair for elective Inguinal Hernias	2012	Willaert W, et al.	Surgical	I-F
Open surgical procedures for incisional hernias	2008	D den Hartog, et al.	Surgical	N-F
Open versus laparoscopic (assisted) ileo pouch anal anastomosis for ulcerative colitis and familial adenomatous polyposis	2009	Ahmed Ali U, et al.	Surgical	I-T
Operative procedures for fissure in ano	2001	Nelson RL, et al.	Surgical	I-T
Oral versus intravenous fluoropyrimidines for colorectal cancer	2017	Chionh F, et al.	Medical	I-F
Oral water soluble contrast for the management of adhesive small bowel obstruction	2007	Abbas S, et al.	Medical	I-F
Palliative chemotherapy for advanced or metastatic colorectal cancer	2000	Best L, et al.	Medical	I-F
Phlebotonics for hemorrhoids	2012	Perera N, et al.	Medical	I-T
Postoperative adjuvant chemotherapy in rectal cancer operated for cure.	2012	Petersen SH, et al.	Medical & Surgical	I-F
Pre and peri-operative erythropoeitin for reducing allogeneic blood transfusions in colorectal cancer surgery.	2009	Devon KM, McLeod RS	Medical & Surgical	N-F
Pre-operative chemoradiation for non-metastatic locally advanced rectal cancer	2012	McCarthy K, et al.	Medical & Surgical	I-T
Pre-operative Nutrition Support in Patients Undergoing Gastrointestinal Surgery.	2012	Burden S, et al.	Surgical	N-T
Prolonged thromboprophylaxis with Low Molecular Weight heparin for abdominal or pelvic surgery	2009	Rasmussen MS, et al.	Medical & Surgical	I-F
Prophylactic nasogastric decompression after abdominal surgery	2007	Verma R, Nelson RL	Surgical	I-F
Propofol for sedation during colonoscopy	2008	Singh H, et al.	Medical & Surgical	I-T
Prosthetic mesh placement for the prevention of parastomal herniation	2018	Jones HG, et al.	Surgical	I-T
Quality of life after rectal resection for cancer, with or without permanent colostomy.	2010	Pachler J, Wille-Jørgensen P	Surgical	N-T
Reconstructive Techniques After Rectal Resection for Rectal Cancer	2008	Brown CJ, et al.	Surgical	I-F
Resection versus no intervention or other surgical interventions for colorectal cancer liver metastases	2007	Fedorowicz Z, et al.	Medical & Surgical	N-T
Rubber band ligation versus excisional haemorrhoidectomy for hemorrhoids	2005	Shanmugam V, et al.	Surgical	I-T
Second**-**line systemic therapy for metastatic colorectal cancer	2017	Mocellin S, et al.	Medical	I-F
Short term benefits for laparoscopic colorectal resection	2005	Schwenk W, et al.	Surgical	I-F
Shouldice technique versus other open techniques for inguinal hernia repair	2012	Amato B, et al.	Surgical	I-T
Single incision versus conventional multi**-**incision appendicectomy for suspected appendicitis	2011	Rehman H, et al.	Surgical	N-F
Single layer versus double layer suture anastomosis of the gastrointestinal tract	2012	Sajid MS, et al.	Surgical	I-T
Stapled versus conventional surgery for hemorrhoids	2006	Lumb KJ, et al.	Surgical	I-F
Stapled versus handsewn methods for colorectal anastomosis surgery	2001	Matos D, et al.	Surgical	I-F
Stapled versus handsewn methods for ileocolic anastomoses	2011	Choy PYG, et al.	Surgical	I-F
Surgical intervention for anorectal fistula	2010	Jacob TJ, et al.	Surgical	N-T
Systemic prokinetic pharmacologic treatment for postoperative adynamic ileus following abdominal surgery in adults	2008	Traut U, et al.	Medical & Surgical	N-T
Transabdominal pre-peritoneal (TAPP) vs totally extraperitoneal (TEP) laparoscopic techniques for inguinal hernia repair.	2005	Wake BL, et al.	Surgical	N-F
Transverse verses midline incisions for abdominal surgery	2005	Brown SR, et al.	Surgical	N-T
Water infusion versus air insufflation for colonoscopy	2015	Hafner S, et al.	Surgical	I-T

*I-F* informs clinical practice*—*firm, *I-T* informs clinical practice*—*tempered, *N-T* does not inform clinical practice*—*tempered, *N-F* does not inform clinical practice*—*firm

## Abbreviations

CCCG: Cochrane Colorectal Cancer Group
I: Informs clinical practice
I-F: Informs clinical practice—firm
I-T: Informs clinical practice—tempered
M: Medical intervention
MS: Medical and surgical intervention
N: Does not inform clinical practice
N-F: Does not inform clinical practice—firm
N-T: Does not inform clinical practice—tempered
O: Other intervention
ROB: Risk of bias
S: Surgical intervention
